# The role of the correlated motion(s) of the chromophore in photoswitching of green and red forms of the photoconvertible fluorescent protein mSAASoti

**DOI:** 10.1038/s41598-024-59364-1

**Published:** 2024-04-16

**Authors:** Alexandra V. Gavshina, Ilya D. Solovyev, Maria G. Khrenova, Konstantin M. Boyko, Larisa A. Varfolomeeva, Mikhail E. Minyaev, Vladimir O. Popov, Alexander P. Savitsky

**Affiliations:** 1 A.N. Bach Institute of Biochemistry, Federal Research Centre ‘Fundamentals of Biotechnology’ of the Russian Academy of Sciences, Moscow, Russia; 2 Department of Chemistry, M.V. Lomonosov Moscow State University, Moscow, Russia; 3 Emanuel Institute of Biochemical Physics, Russian Academy of Sciences, Moscow, Russia; 4 N.D. Zelinsky Institute of Organic Chemistry Russian Academy of Sciences, Moscow, Russia

**Keywords:** Biphotochromic fluorescent proteins, Green-to-red photoconversion, Reversible photoswitching, Photochemistry of fluorescent proteins, Crystal structure, Dynamic network analysis, Biophysical chemistry, Biological fluorescence, Protein design

## Abstract

Wild-type SAASoti and its monomeric variant mSAASoti can undergo phototransformations, including reversible photoswitching of the green form to a nonfluorescent state and irreversible green-to-red photoconversion. In this study, we extend the photochemistry of mSAASoti variants to enable reversible photoswitching of the red form. This result is achieved by rational and site-saturated mutagenesis of the M163 and F177 residues. In the case of mSAASoti it is M163T substitution that leads to the fastest switching and the most photostable variant, and reversible photoswitching can be observed for both green and red forms when expressed in eukaryotic cells. We obtained a 13-fold increase in the switching efficiency with the maximum switching contrast of the green form and the appearance of comparable switching of the red form for the C21N/M163T mSAASoti variant. The crystal structure of the C21N mSAASoti in its green on-state was obtained for the first time at 3.0 Å resolution, and it is in good agreement with previously calculated 3D-model. Dynamic network analysis reveals that efficient photoswitching occurs if motions of the 66H residue and phenyl fragment of chromophore are correlated and these moieties belong to the same community.

## Introduction

Photoswitchable, photoconvertible and multi-transformable proteins are fine tools for multimodal measurements using super-resolution microscopy^1–3^. SAASoti fluorescent protein (FP) was first described in^4^; later, the monomeric form—V127T or mSAASoti—was obtained, and its applications in the PALM method^5^ and FCS study of enzymatic activity in a single live cell^6^ were successfully demonstrated. From here on, the numbering of all amino acid residues is given according to the SAASoti sequence^4^.

Reversible photoswitching often proceeds via the *cis–trans* isomerization of the chromophore, associated with the change in its protonation state and plane distortions of the π-conjugated system^7–9^. Photoconversion is an irreversible photochemical process of peptide chain breaking, leading to the formation of a new π-conjugated system and a redshift of the spectral parameters^10,11^. For most photoconvertible proteins chromophore undergoes photoconversion in the protonated state during violet light (400 nm) absorption, but in the case of Dendra2 photoconversion is also possible at a wavelength of 480 nm^12^, also resulting in the β-elimination reaction in the chromophore forming amino acid triplet –HYG–.

As original mSAASoti has both photoconversion^4^ and photoswitching properties^5,13^, it can be referred to as a biphotochromic fluorescent protein. However, in the case of mSAASoti, only the green form can be switched-off to the dark state^14^. The first strategies for biphotochromic protein production^15^ were based on the experience with Dronpa protein^16,17^. By means of random mutagenesis, Ando et al.^17^ determined the most relevant residues in the chromophore vicinity—V161, M163 and F177, and their replacement led to an increase in the efficiency of switching to the off-state when irradiated with excitation light. As a result, Dronpa-2 (M163T) and Dronpa-3 (V161I/M163A) variants were obtained, and the M163T variant showed greater photoswitching efficiency. A single substitution V161G is sufficient to increase the switching rate in the case of rsFastLimeFP^18^. The F177C, V161G, M163C and a number of other substitutions resulted in the bsDronpa protein^19^. At the same time, the switched-off bsDronpa has a more long-lived dark state, which is confirmed by long relaxation times to the on state compared to single amino acid substitutions. The data obtained at different setups are difficult to compare, but using the example of the proteins in the Dronpa family^17–19^, we can conclude that the most effective photoswitching to the dark state was observed for M163T Dronpa. The first biphotochromic protein—IrisFP—was obtained by random mutagenesis in the EosFP gene, and contained the F177S substitution^20^, which is similar to the strategies described above. In addition to the rearrangement of the close a.a. environment, a strong relocation of the I161 side chain was noted during photoswitching as it was revealed during the structural analysis of IrisFP^20^. Rational mutagenesis was performed on mEosFP and Dendra2. The impact on I161 in the case of Dendra2 did not lead to significant changes.

In the case of mSAASoti the corresponding a.a. residues occupy the same positions (Table S1). Therefore, the strategy of replacing amino acid residues in positions 163 and 177 to increase the switching efficiency of both green and red forms should be considered for an improvement of the mSAASoti photoswitching.

## Results

**Figure 1:**
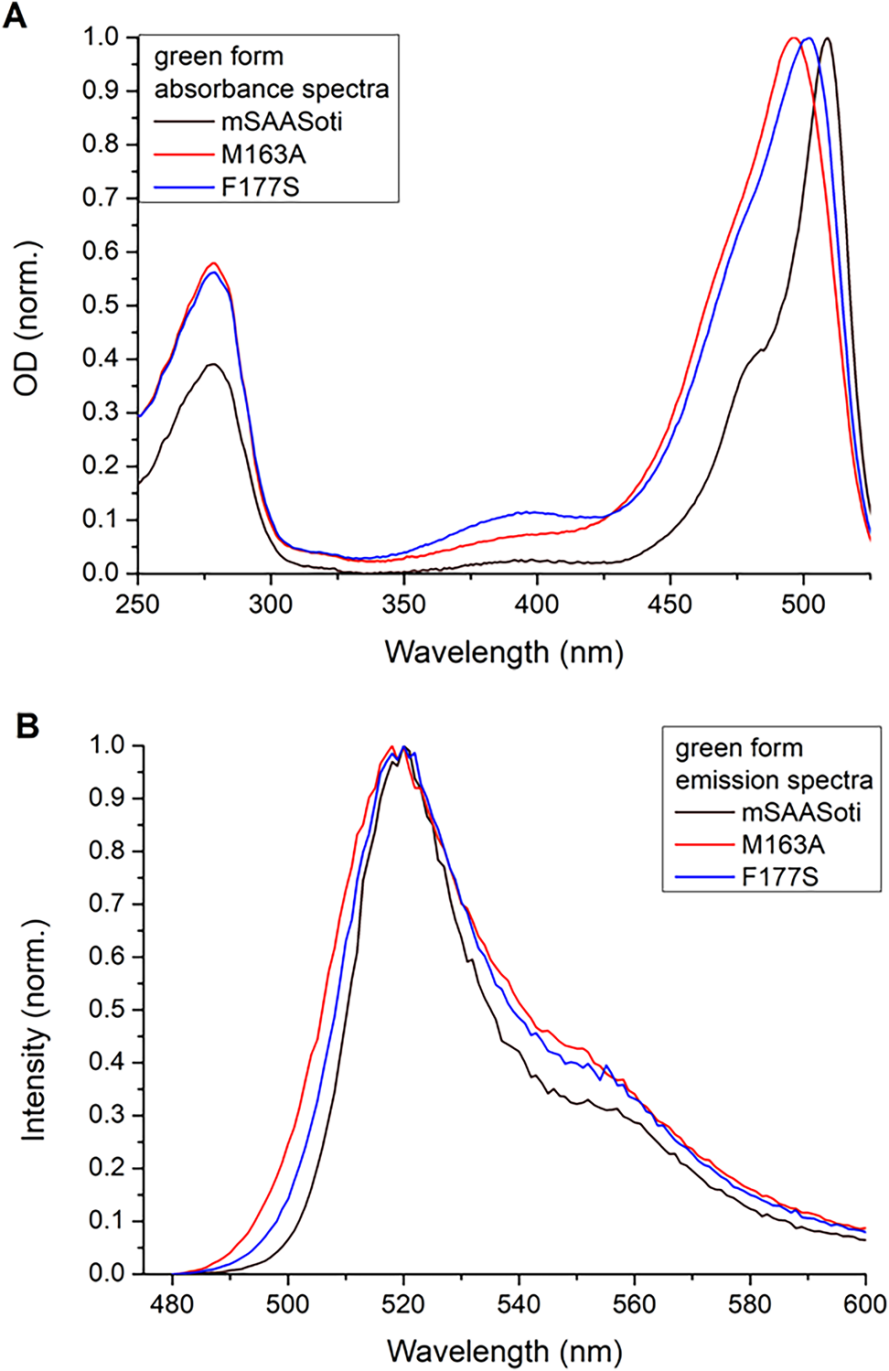
Normalized absorption (**A**) and emission (**B**) spectra registered for different mSAASoti mutants in 20 mM Tris–HCl, 150 mM NaCl, pH 7.4 buffer measured on Cary 60 and Cary Eclipse, respectively.

We carried out rational mutagenesis to obtain M163A and F177S mSAASoti mutant forms based on the experience of obtaining biphotochromic proteins^15,20^. After protein isolation and purification, we characterized their spectral and physicochemical properties (λex/λem, ε, pKa). Both of these mutants were shown to have a blueshift of the absorption maxima (Fig. 1A), while the emission maxima remained unchanged (Fig. 1B).

The extinction coefficients at neutral pH decreased, which may be caused by the pKa shift to a more alkaline region (Table 1). As it can be seen in the absorption and emission spectra (Fig. S3), 400 nm illumination during 10 min results in the appearance of the ~ 560 nm absorbance and ~ 580 nm emission bands corresponding to the red form, but the green form is still present in the solution, which is also confirmed by the SDS-gel electrophoresis and the incomplete peptide chain cleavage (Fig. S4). The lower fluorescence intensity of the red form for the mutants with substituted M163 and F177 residues is due to the fact that most of the red form is in the protonated state, and has a low quantum yield value compared to the wild type protein (Fig. S3B,D,F). Fluorescence quantum yield (φ) of the new mutants turned out to be 2 times smaller for the green forms, whereas φ values of the red forms did not much change in comparison with the wild type mSAASoti (Table 1). In addition to spectral changes, there were changes in the fluorescence lifetime; a slight decrease from 3.3 to 3.0 ns was observed for green F177S, and a bi-exponential dependence of fluorescence attenuation with lifetimes of 2.4 and 1.2 was observed for green M163A.

**Table 1:** Spectral properties and QY of the obtained mSAASoti mutant forms.

Mutant form	Green form λex/λem, nm	Red form λex/λem, nm	ε (G/R) /1000 M^−1^*cm^−1^	QY (φ) Green/Red	pKa G/R
mSAASoti ^5^	509/519	578/589	75/24	0.59/0.27	6.3/6.6
C21N ^21^	509/519	579/590	82/25.4	0.61/0.26	6.4/7.5
M163A	496/519	565/587	62/0.3*	0.30/0.24	6.7/7.5
M163T	498/515	565/582	56/37	0.26/0.24	6.6/7.4
C21N/M163A	497/517	561/587	53/18	0.27/0.25	6.5/7.3
C21N/M163T	498/516	565/580	53.5/12.6	0.26/0.25	5.9/7.2
C21N/M163G	496/516	558/581	50/5.3*		5.6/7.0
F177S	501/519	565/588	51/1*	0.48/0.23	6.8/7.8
C21N/F177A	503/518	566/588	42/3*		6.3/7.7
C21N/F177N	503/517	564/587	55/4*		6.2/7.8
C21N/F177T	506/518	573/588	66/4.5*		6.2/7.6
C21N/F177S	502/518	568/587	47/3*		6.7/7.7

*Apparent value that does not take into account the photobleaching process during green-to-red photoconversion under 400 nm illumination.

As in the case of other proteins of this group, positions 163 and 177 turned out to be sensitive to on-to-off photoswitching for mSAASoti, but their substitutions to alanine and serine greatly affected the red form (significant pKa shift, small extinction coefficient, Table 1). The example of pKa calculation is presented on Fig. S1 and Eq. (S1). For this reason, we conducted site-saturation mutagenesis using degenerate primers and obtained M163X, F177X, C21N/M163X and C21N/F177X mSAASoti variants (X indicates any amino acid residue). As it was shown in the previous study^21^, C21N substitution abolished a dimeric fraction of the mSAASoti at higher concentrations and resulted in the true monomeric protein. After the cloning BL21 (DE3) *E. coli* cells were transformed with the ligation products, and the cells were seeded on Petri dishes covered with LB agar. Fluorescent screening of the colonies was conducted using the homebuilt equipment (Fig. S2), the colonies were illuminated with 470 and 400 nm light, while the emission spectra were recorded over time. The most promising colonies were sequenced to determine the appropriate substitution. Among them, C21N/M163A, C21N/M163G, C21N/M163T and C21N/F177A, C21N/F177N, C21N/F177T, C21N/F177S mSAASoti proteins were also isolated and purified. As it can be seen from Table 1, sub-group with the F177X substitution has a significant shift of the pKa values for the red form.

Only the green form of the wild-type mSAASoti protein can be photoswitched to the dark state^14^, whereas in the case of M163X and F177X mSAASoti variants, we observed photoswitching of both green and red forms. To analyze the on-to-off photoswitching, we irradiated colonies expressing different mSAASoti mutants with 390, 485 and 550 nm light, after the preliminary fluorescence screening, we determined mutants with the maximum photoswitching rate—C21N/F177S, C21N/M163T, C21N/M163A and C21N mSAASoti as a reference.

### The origin of absorption spectra variations

**Figure 2:**
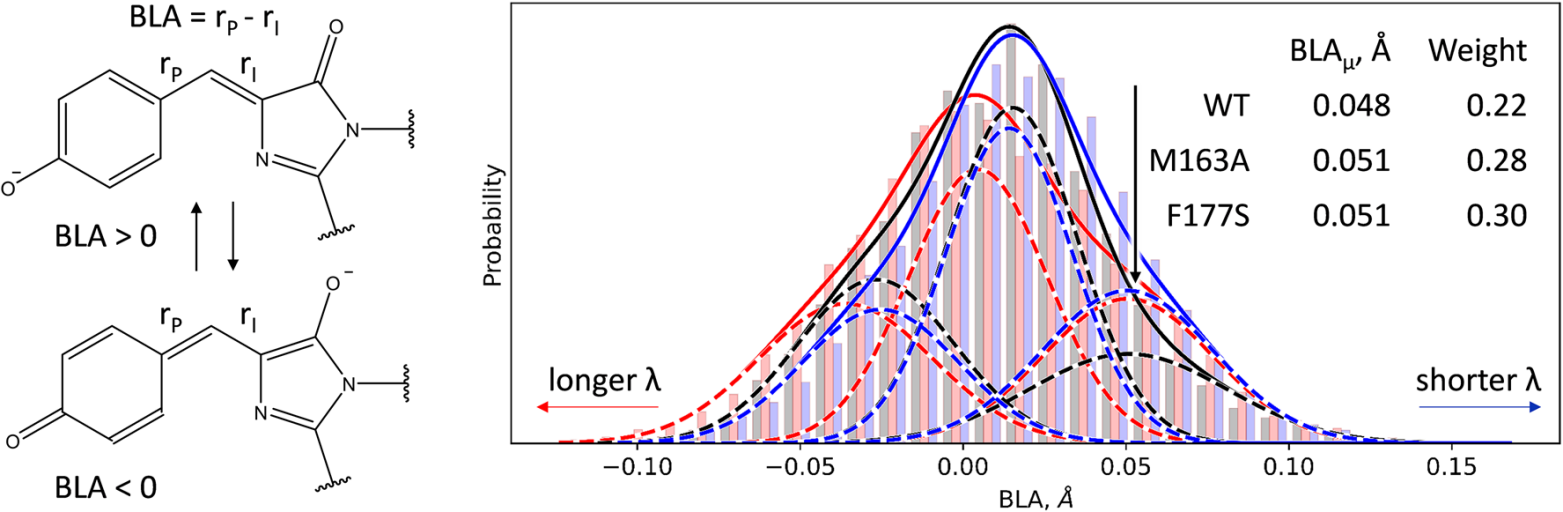
Bond length alternation (BLA) in the GFP-type chromophore and its distributions in the green anionic form of mSAASoti (black bars and lines) and its M163A (red bars and lines) and F177S (blue bars and lines) variants. Fitting of each distribution is performed for three Gaussians; weights and mean values corresponding to the most right shifted component for each model are shown and marked with an arrow.

We performed molecular dynamic simulations with QM/MM potentials to explain the origin of the broadening of absorption band of M163A and F177S mSAASoti variants compared with mSAASoti. It was already shown for fluorescent proteins with GFP-type chromophore that the value of bond length alternation (BLA, difference between lengths of C–C bonds in the C–C=C bridge of the chromophore, Fig. 2) correlates with the maximum of the absorption band^22,23^. Structures with a predominance of the resonance form with BLA > 0 are characterized by a larger energy gap between the ground and excited electronic states and a shorter wavelength of transition. We deduce that the distribution of BLA should correlate with the absorption band shape. To study this, we performed QM/MM MD simulations of three model systems, mSAASoti and its M163A and F177S variants. BLA distributions are shown in Fig. 2. The main difference is observed for the weights of the fractions characterized by larger BLA values and consequently corresponding to the shorter wavelengths in the absorption spectrum. The overall distribution is wider for mSAASoti M163A and F177S, which is also in agreement with experimental observations. Thus, changes in the absorption band shape can be explained by the overall change in the influence of the entire protein on the chromophore group pronounced in the change in distribution between two resonance forms of the negatively charged chromophore. We can speculate that the fraction with the positive BLA with the predominance of the phenolic form (black arrow on Fig. 2) is responsible for the more efficient cis–trans isomerization, that is experimentally observed for the M163A and F177S compared with the mSAASoti.

### Tagging with mSAASoti mutants

**Figure 3:**
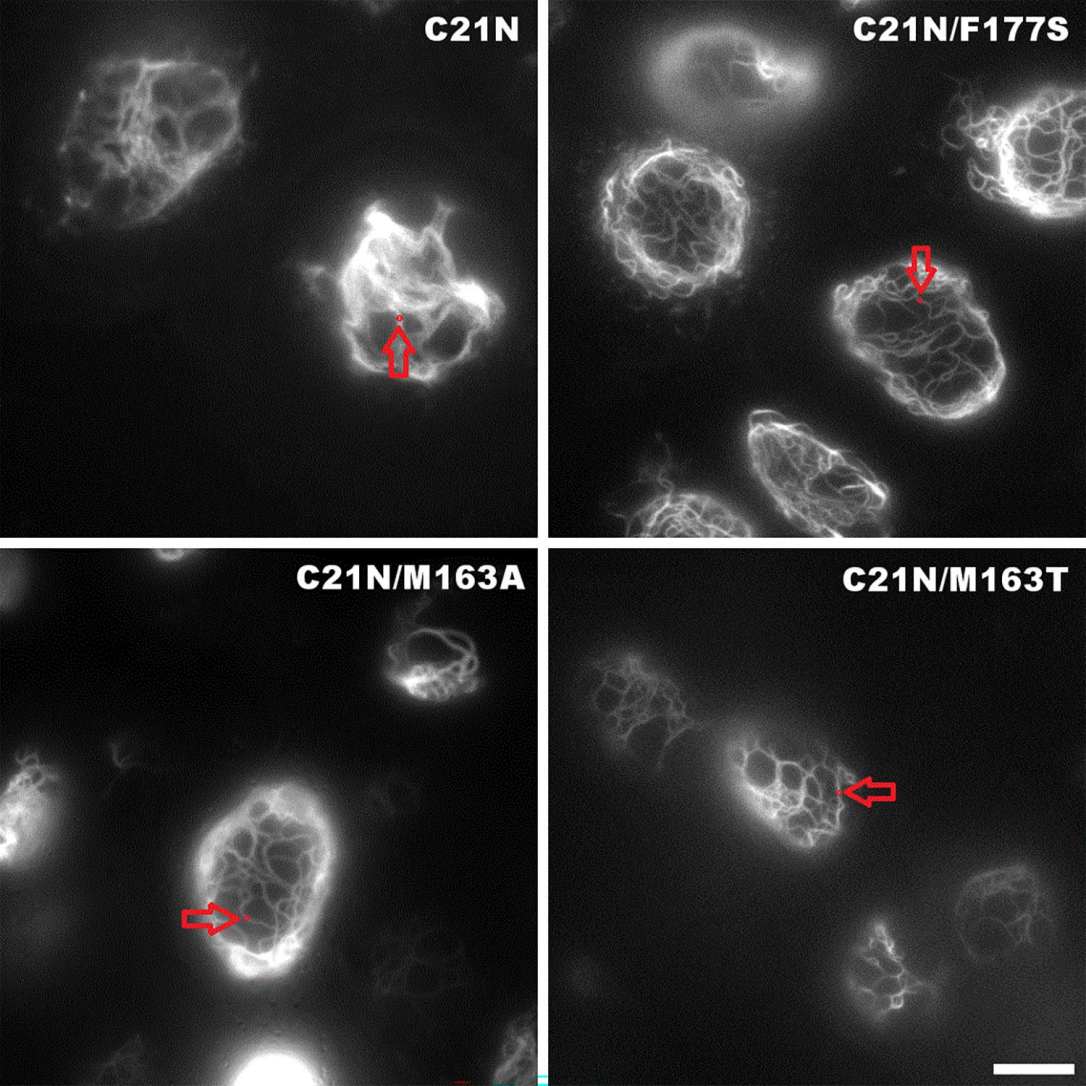
Different mSAASoti fusions with vimentin expressed in HeLa Kyoto cells, 24 h after transfection. Scale bar, 10 μm. The images were acquired in the green channel. The fluorescence intensity for the kinetic measurements of the reversible photoswitching and photoconversion (described below) was studied at the points indicated by red arrow.

C21N, C21N/M163T-, C21N/M163A, and C21N/F177S mSAASoti mutant forms were cloned to vimentin genes into the pcDNA3 vector and expressed in human HeLa Kyoto cells. The images were recorded 24 h post transfection (Fig. 3).

### On-to-off reversible photoswitching

To compare the switching behavior between different mSAASoti mutants in eukaryotic cells we studied on-to-off switching cycles using sequential illumination of the cells expressing mSAASoti-vimentin gene constructions described above. The green form was illuminated with 485 nm light to realize on-to-off switching and the on-state was regenerated by 390 nm pulses attributed to the absorption of protonated state of the green chromophore (Fig. S5A,C). The red form was generated by 390 nm illumination, switched-off with 548 nm light and switched-on by 434 nm pulses attributed to the absorption of the red protonated state of the chromophore (Fig. S5B,D). The decrease of fluorescence signal obeys a mono-exponential law for both green and red forms.

1 $$ I\left( t \right) = A*\exp \left( { - k*t} \right) + c $$

**Figure 4:**
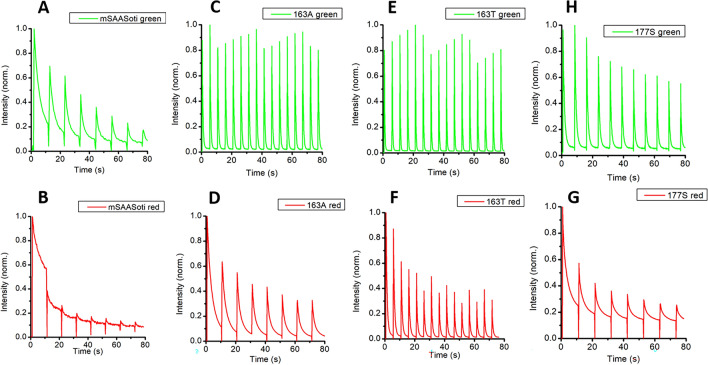
Photoswitching kinetics in eukariotic cells (HeLa Kyoto), expressing different mSAASoti-vimentin variants: C21N (**A**, **B**), C21N/M163A (**C**, **D**), C21N/M163T (**E**, **F**), C21N/F177S (**G**, **H**)). On-to-off PS of the green form was carried out using 485 nm light (10 s) with subsequent activation by 390 nm light (0.5 s). The red form was generated by 390 nm illumination during 5 s, switched-off by 548 nm (10 s), and activated by 434 nm light. The kinetic curves were plotted based on emission maxima for both forms, off-to-on activation exposure is shown with a vertical line. The data is normalized to the initial fluorescence intencity for both forms.

As it can be seen from Fig. 4 and Table 2, the maximum photoswitching rate for both forms were observed for C21N/M163T variant along with the maximum switching contrast (98% and 97% for the green and red forms, respectively). Interestingly, the red form of M163A switches off more slowly, than that of M163T, but their green forms switch with comparable rates. C21N/M163T mSAASoti switches to the off state 13 times faster than C21N (Table 2).

**Table 2:** Kinetic parameters (k_off_) and switching contrast (SC, %) in the reaction of on-to-off photoswitching according to Eq. (1).

	Green (k_off_, s^-1^)	SC, %	Red (k_off_, s^-1^)	SC, %
C21N	0.27 ± 0.01	83	–	–
C21N/M163A	2.31 ± 0.08	97	0.37 ± 0.05	92
C21N/M163T	3.39 ± 0.34	98	1.45 ± 0.06	97
C21N/F177S	1.16 ± 0.07	94	0.37 ± 0.05	77

We also studied repeated on–off cycles for the green and red forms of these mSAASoti mutants. *Green form photoswitching.* During the cycles of sequential photoswitching with 485 nm light with the regeneration of the fluorescent form with 390 nm light of the HeLa cells expressing different mSAASoti mutants in ORF with vimentin gene, it was found that M163A and M163T substitutions led to more photostable variants, which is apparently due to the exclusion of the photooxidation stage of methionine (Fig. 4)^14^, whereas the initial fluorescence intensity decreases markedly from cycle to cycle in the case of F177S/C21N and C21N mSAASoti variants. M163A/T variants also demonstrated the maximum switching contrast of the green form. *Red form* was generated by 390 nm illumination of the cells required for green-to-red photoconversion, after that 548 nm light was used to photoswitch between red fluorescent and red dark states, and 434 nm light was used to regenerate the fluorescent red form. As for red forms C21N/M163T variant has the maximum photoswitching rate and contrast among the mutants. Interestingly, that the red form of C21N/M163A differs significantly from C21N/M163T. Thus, red C21N/M163A switches more slowly to the dark state with the reduced switching contrast (Fig. 4D,F). Illumination with 548 nm light of the red form of C21N mSAASoti led only to photodestruction with minimal photoswitching (Fig. 4B).

## Crystal structure of the mSAASoti

**Figure 5:**
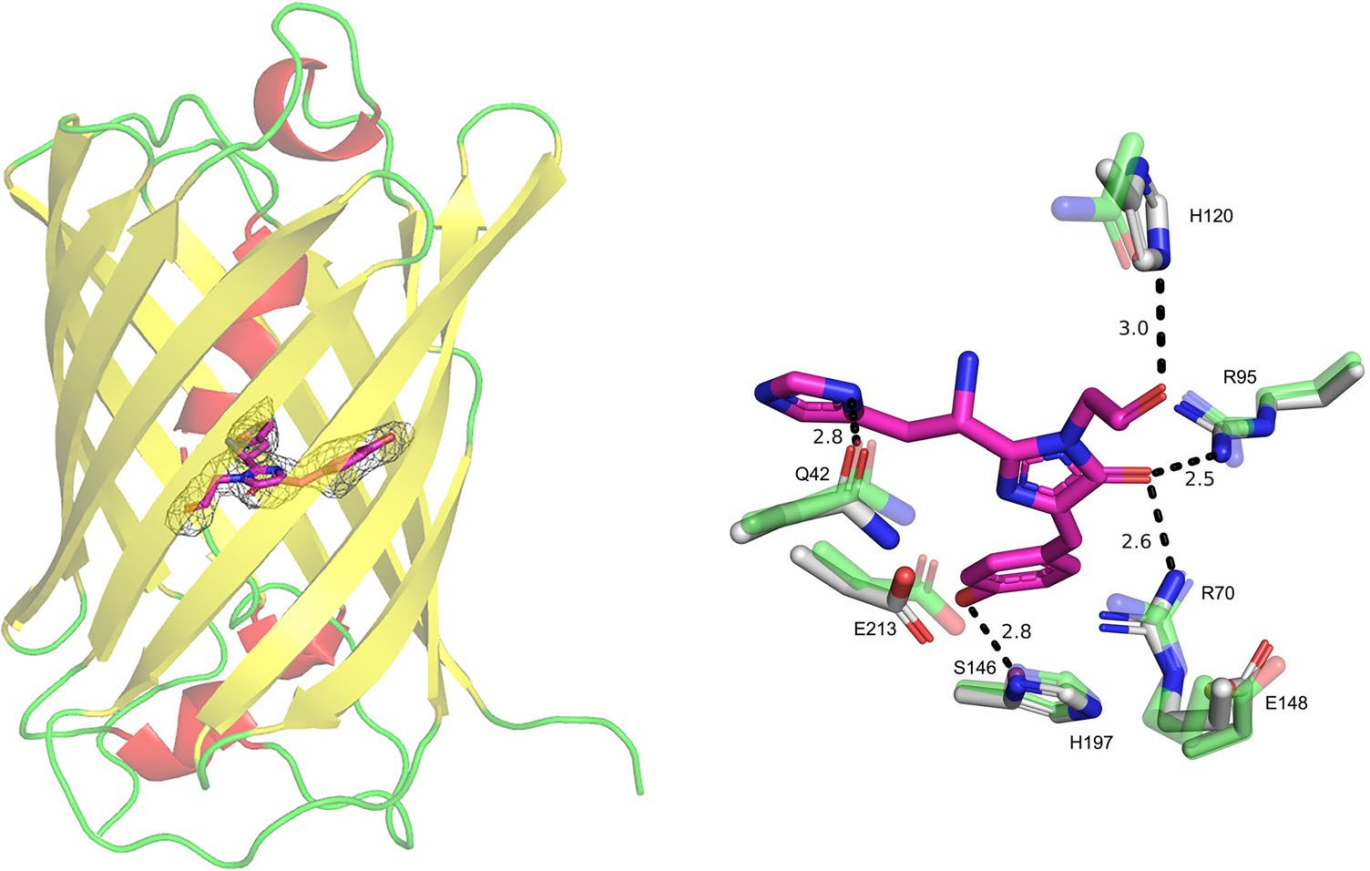
C21N mSAASoti structure. (Left) mSAASoti is colored by secondary structure elements and shown semi-transparent for clarity. Polder map for the chromophore at 3σ level is shown as gray mesh. (Right) Superposition of C21N mSAASoti (white sticks) and IrisFP, PDB-code 2VVH, (green translucent). The amino acid residues in the chromophore vicinity are indicated according to mSAASoti numbering. Hydrogen bonds are depicted as black dashed lines, corresponding distances are labeled.

The crystal structure of the C21N mSAASoti variant in its green on-state was obtained at 3.0 Å resolution. The analysis of crystal contacts demonstrated that SAASoti is a monomer supporting biochemical data^5,21^. The mSAASoti has a typical β-barrel fold with the chromophore ^66^HYG^68^ resided on the central α-helix (Fig. 5). In spite of the moderate resolution, the electron density clearly revealed a maturated chromophore in its cis-conformation as well as conformation of nearby residues. The chromophore is fixed with a number of hydrogen bonds with neighboring amino acid residues. The oxygen of the chromophore carbonyl group is hydrogen bonded to side chain of H120, while oxygen of imidazolidone moiety is fixed with side chain of two arginine residues—R70 and R95. Histidine group of the chromophore forms hydrogen bond to side chain of Q42. Finally, the OH-group of the tyrosine moiety is hydrogen bounded to side chain of S146. Noteworthy, the orientation of the chromophore’s tyrosine ring is stabilized through stacking interaction with side chain of H197. The superposition of C21N mSAASoti and IrisFP (PDB-code 2VVH) structures demonstrated similar conformation of the residues coordinating the chromophore (Fig. 5, Right).

### The origin of photoswitching in fluorescent proteins

**Figure 6:**
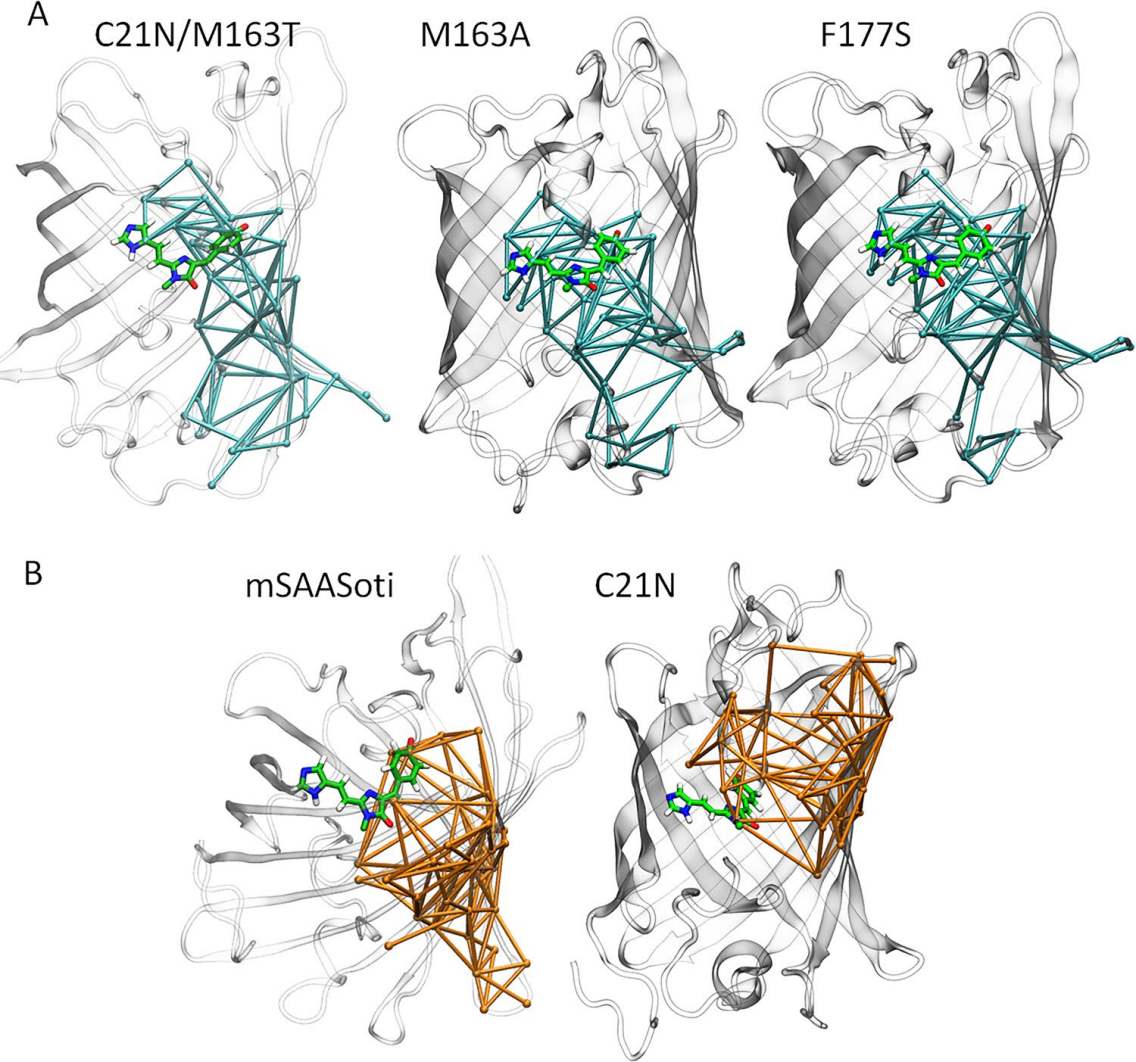
Communities comprising phenyl fragment of the red form of the chromophore in different variants if the mSAASoti. (**A**) For variants with the experimentally observed photoisomerization of the red form the phenyl fragment of the chromophore and the former side chain of His66 belong to the same community (shown in cyan sticks). (**B**) For non-isomerizing variants these two fragments belong to different communities (communities with the phenyl fragment are shown in orange). Color code: carbon—green, oxygen—red, nitrogen—blue, hydrogen—white.

We performed dynamic network analysis to determine the features that are responsible for *cis–trans* chromophore isomerization. To do this, we obtained a set of models with both green and red forms of the chromophore in the anionic *cis*-form for mSAASoti and its C21N, C21N/M163T, M163A, and F177S variants. We simulated 300 ns trajectories for each model system. For all considered systems, photoswitching is observed for the green form, and in the red form, it is observed only in variants with substitutions at either the 163 or 177 positions. Dynamic network analysis revealed groups of residues with correlated motion, and we determined the residues that belonged to the same group as the phenyl fragment of the chromophore (Fig. 6). According to our calculations, the systems that can undergo isomerization have a distinctive feature: the phenyl fragment of the chromophore and the former H66 residue that is the part of the red chromophore belong to the same community. Importantly, this criterion works for both red and green forms despite the considerable differences in this region. In red form, both π-systems of the H66 residue side chain and a green form of the chromophore are united to the extended conjugated π-system.

We examined neighboring residues that may be responsible for the ability to isomerize. However, we found no similarities between the considered systems in this respect. Even in structurally similar mSAASoti and its C21N variant residues located close to the phenyl part of the chromophore, F177, M163 and I161 act differently. In red form, the motion of the phenyl part of the chromophore is correlated with F177 in mSAASoti and with M163 and I161 in its C21N variant. Similarly, involvement of M163 and I161 is observed for the F177S variant and is not observed for the species with substitution at the 163^rd^ position.

## Discussion

For all mSAASoti mutants studied we observed significant variations in physical and chemical properties, first of all pK-shift of the green and red forms, absorption spectra, on-to-off photoswitching and recovery to the fluorescent state, photochemical stability.

The pKa values of M163X and F177X mSAASoti mutants were found to be more alkaline for both green and red forms (Table 1), whereas M163A substitution in the case of EosFP^15^ strongly shifts the pKa (4.3 instead of 5.3) of the green form to the acidic pH, which is apparently the reason for the loss of the photoconversion ability. As studied earlier, C21N mSAASoti had an extremely high pKa (7.5) of the red form; however, this amino acid residue is oriented outside the β-barrel, and the reason for the phenomenon was described in^21^. Values greater than 7.5 significantly reduce the number of fluorescent molecules in the cytoplasm, and the intensity becomes sensitive to small changes in the local pH, but all the mSAASoti mutants with the substituted F177 have extremely low extinction coefficients and very high pKa. Interestingly, the addition of C21N substitution to M163X and F177X mutants resulted in the lowering of pKa (Table 1) in comparison with single M163 and F177 mutants. M163- and F177-substituted variants of EosFP and Dendra2^15^ as well as mSAASoti mutants have blueshifted emission spectra. For M163A and F177S mSAASoti mutants, we observed a decrease in the fluorescent lifetime values, as described earlier for M163A and F177S mEosFP and Dendra2^15^. The biexponential nature of the fluorescence decays was observed for the M163/F177 mutants of mEosFP and Dendra2, whereas the lifetimes of the original proteins were higher and the fluorescence decay obeyed the monoexponential law. The biexponential dependence of the fluorescence decays may be associated with the presence of various radiating conformers, probably formed as a result of the special mobility of the chromophore. Molecular dynamic simulations with QM/MM potentials revealed that broadening of the spectra in the case of new mutants can be explained by the change in distribution between two resonance forms of the negatively charged chromophore.

The decrease in the fluorescence quantum yield is probably associated with increased flexibility of the chromophore, as described earlier^15^, but in the case of SAASoti it decreased more dramatically for the green form.

M163T substitution in the case of C21N mSAASoti led to a greater acceleration of the on-to-off photoswitching with the maximum switching contrast for both green and red forms, whereas the protein remained photoconvertible to the red form. M163 and F177 substitutions contributed to the appearance of photoswitching of the red form without preliminary irradiation of the green form with blue light, as it was previously found for original mSAASoti^14^. It is worth noting that for the photoconvertible variant of pcDronpa, only the green form of the protein is subjected to photoswitching ^24^. In the case of mEosFP and Dendra2, the F177S substitution led to the fastest photoswitching variants for both green and red forms and resulted in the most promising fluorescent markers (IrisFP and NijiFP, respectively). Dreiklang is an example of a protein with a completely different switching mechanism. Dreiklang FP has a distinct switching mechanism—chromophore hydration instead of cis–trans isomerization, which entailed a change in the switching wavelengths: 365 nm—on, 405 nm—off, and the excitation maximum 511 nm^25^.

Substitutions of M163 led to increased photostability when photoswitching was repeated. Phototransformations leading to a decrease in fluorescence intensity from cycle to cycle are also associated with methionine 163 photooxidation^14^, and its replacement allows more switching cycles to be conducted without a decrease in the total fluorescent signal. It is worth noting that the result of M163 oxidation is also an increase in the switching rate of the green SAASoti form^21^ and the appearance of minor photoswitching of the red form^14^. Methionine oxidation and increased protein stability as a result of its replacement were also noted for other biphotochromic proteins when irradiated with low power light^26,27^.

Many photoconvertible fluorescent proteins have a weak photoswitching ability^15,28^; however, it is many orders of magnitude inferior in the case of biphotochromic proteins^15^ and comparable in rate constants to irreversible photodestruction. The restricted chromophore mobility makes *cis–trans* isomerization difficult and may look like intensity jumps with low contrast. Thus, it must be noted that we observed a small switching amplitude of the red SAASoti form, generated with 470 nm illumination of the green form prior to photoconversion, and the acceleration of the photoswitching of the green form. Later, M163 oxidation was confirmed by mass spectrometry^14,21^. On the other hand, a large switching amplitude was observed, particularly for the red form in the case of mMaple, and an increased amount of protonated red chromophore was recorded after irradiation with green light in the absorption spectra^28^.

The crystal structure of the C21N mSAASoti in its green on-state was obtained for the first time at 3.0 Å resolution, and it is in good agreement with previously calculated 3D-model. Structural alignment with the IrisFP structure (2VVH) revealed that residues E148, R70, H197, E212 (numbering according mSAASoti) occupy the same position around the chromophore. It is interesting, that both conservative triad Glu144 − His194 − Glu212 ‘catches’ the *cis* chromophore configuration and Glu144 − Arg66 − Glu212 triad—in the *trans*-configuration in IrisFP^29^ occupied the same position in mSAASoti (Fig. 5). Using dynamic network analysis, we demonstrated for the first time that the ability of the chromophore to isomerize in both red and green forms is governed by the specificity of motion of its phenyl fragment. If its motion is correlated with the motion of H66 and these fragments belong to the same community, isomerization may occur. Importantly, this conclusion is valid despite the different chemical structure of the red and green chromophores.

## Methods

### Plasmid design

Site-directed and site-saturated mutagenesis were performed by the overlap PCR method, as described in^5^, primer synthesis, including degenerate primers, was carried out by Evrogen, LLC.

C21N, C21N/F177S, C21N/M163A and C21N/M163T mSAASoti genes were amplified, digested with EcoRI and XbaI and ligated with similarly digested pVimentin-SAASoti, obtained and described earlier^5^. mSAASoti genes for in vitro characterization were cloned into pET22b vector, while vimentin fusion constructs were cloned into pcDNA3 vector for mammalian expression.

### Cell culture

HeLa Kyoto cell line was a kind gift from Dr. Alexey M. Bogdanov and grown in DMEM high glucose medium (PanEco), containing 5%FBS, 0.5% (v/v) penicillin–streptomycin (PanEco) and at 37 °C in a humidified 5% CO_2_ atmosphere. The cells were transfected with a GenJect™-39 reagent (Molecta) according to the manufacturer’s protocol.

### Colony screening

The ligating mixture after overlap PCR with degenerate primers was transformed into *E. coli* BL21(DE3) cells by electroporation and seeded on agar LB medium with the addition of 100 μg/ml ampicillin. The induction of expression occurred due to the natural "leakage" of the lac operon, and the addition of IPTG did not affect the expression of mSAASoti variants. Bacteria expressing different mSAASoti mutants were grown at 20° C on Petri dishes, and colonies expressing fluorescent proteins were observed the next day. After that, the colonies were analyzed by spectral and kinetic switching properties. After screening, PCR of promising colonies was performed, and the fragments were transferred to LLC Evrogen for sequencing. At the final stage, 3 new forms of M163X and F177X were selected, and the proteins were refined and purified according to the method described in^14^. Further analysis of the purified preparations was carried out in buffer solutions.

### Absorbance and fluorescence measurements

Absorbance spectra were registered using a Cary 60 (Agilent, USA) spectrophotometer and fluorescence spectra were registered using a Cary Eclipse (Varian, Australia) fluorescence spectrophotometer at the room temperature (22 °C) in a 3 mm quartz cuvette (Hellma, Germany). The devices use a pulsed xenon lamp with low radiation intensity; therefore, we minimize the effect of light on the sample when registering the spectra. Molar extinction coefficients were determined in solutions of purified mSAASoti samples in the 20 mM Tris–HCl, 150 mM NaCl, pH 7.4 buffer from the absorption maxima of green and red forms, taking into account theoretically calculated values of ε280 (https://web.expasy.org/protparam/), A280 (by absorption spectrum). The red form of the SAASoti was obtained by irradiation with 390 nm light at a low dose and it was assumed that photodestruction did not occur. In this case, the extinction coefficient of the red form was calculated as the ratio of the increase in absorption of the red form to the decrease in absorption of the green form multiplied by the extinction coefficient of the green form. For values marked * in Table 1, samples were irradiated until the maximum optical density of the red anionic form was obtained. The obtained value was taken as the extinction coefficient without taking into account the photodestruction. The relative quantum yield of the new mSAASoti mutant forms was determined in comparison with the previously determined value for mSAASoti, as it had been measured using the absolute, calibration-free nanocavity-based method ^5^. To do this, 6 samples of solutions with an optical density at an excitation wavelength not exceeding 0.1 were prepared for each mutant form and for mSAASoti. For each sample, the absorption spectra and the corrected fluorescence spectra were recorded. The quantum yield was determined by the ratio of the dependences of the areas under the fluorescence spectra to the optical density of the sample.

### Spectral and phototransformation properties characterization setup

The selection of promising clones expressed in *E. coli* colonies after site-saturated mutagenesis was carried out on an epifluorescence installation with a spectrometer. Solutions of isolated and purified proteins of new mutant forms were analyzed in a capillary using the same colony screening installation.

We used a homemade spectroscopic setup to analyze the colonies and small volumes of the solutions (SI, Fig. S2). An Olympus BX-43 body was used as the base of the device. Four Thorlabs LEDs were collimated by the achromatic condenser lenses Thorlabs ACL2520-A and coupled by the 3 dichroic mirrors Thorlabs DMLP425R, DMLP490R and Edmund Optics #67–078, 458 nm long pass. Spectral bands were carried out by the bandpass filters Thorlabs MF390/18 and Chroma ET448/19x, ET470/24 m, ZET561/10 × or ET560/25x. We used the Köhler scheme to obtain a more homogenous light beam after the microscope objective. The light beam after collimating lenses was focused on the objective back plane by the achromat lens (Thorlabs AC254-125-A). Then, light after achromat was reflected by the 50/50 beam splitter on the objective. The fluorescence image was projected on the CCD camera after beam splitter trough tube lens after Chroma 500LP and ZET562NF Notch filter. A camera is used to focus and orient the sample. At the same time, an achromatic lens focused the image on the entrance slit of the Avesta ASP-75 spectrometer through the second 70/30 beam splitter. LEDs were controlled by the Thorlabs LEDD1B driver and homemade USB DAC with self-written Python software. It allows switching LEDs with 1 ms time resolution. We obtained 282.4, 528.4, 706.1, and 45.6 (ZET561/10x) mW/cm^2^ maximal light power densities for 390, 450, 470 and 560 nm, respectively, after 20x/0.4 NA Olympus PlanApo objective.

Colonies and purified proteins of all other variants indicated in Table 1 were irradiated with 470 nm light with a power density of 437 mW/cm^2^ (green form, on-to-off switching), 400 nm 148 mW/cm^2^ light (green-to-red photoconversion, off-to-on switching), 560 nm 45 mW/cm^2^ to (red form, on-to-off switching) and 450 nm 305 mW/cm^2^ (red form, off-to-on switching).

Kinetic data analysis was carried out as previously reported^21^.

### Microscopy and phototransformation kinetics

We obtained the cell images and phototransformation kinetics of vimentin-SAASoti variants using Olympus IX-71 microscope with × 100, 1.49 NA objective. For excitation we used Lumencor SpectraX light engine with 390/22 nm (10%—1.4 mW/cm^2^ before objective), 434/17 nm, 485/25 nm (10%—2.9 mW/cm^2^), 548/10 nm (20%—5 mW/cm^2^) excitation wavelength, U-MWB2 filter cube in the green and U-MWG2 in the red channel. For acquisition we used Andor iXon 888 EMCCD camera.

### Molecular modeling

The 3D model of mSAASoti was obtained in^21^ and utilized in this study. Careful comparison of the 3D model with the experimental structure obtained in this study demonstrate that those are in agreement with each other. Amino acid substitutions were performed in this model to obtain mSAASoti variants. The CHARMM36^30^ force field parameters were utilized for protein and the CGenFF^31^ force field parameters for the chromophore in the green form. The system was solvated in a rectangular water box with TIP3P^32^ water molecules and neutralized by adding sodium ions. Classical molecular dynamics simulations were performed in the NAMD software package^33^. Each system was preliminarily equilibrated by 10,000 minimization steps and a 20 ns MD run. Production runs were performed for 400 ns. All simulations were performed with a 1 fs time step in the NPT ensemble at p = 1 atm and T = 300 K. The pressure and temperature were controlled by a Nosé-Hoover barostat and Langevin thermostat, respectively. The cutoff distances were 12 Å for both electrostatic and van der Waals interactions with switching to the smoothing function at 10 Å. Dynamic network analysis^34^ was utilized to dissect communities in which motions are correlated. According to this approach, a network is defined as a set of nodes connected by edges. In this work, every amino acid was represented by a single node; the chromophore was divided into three nodes. Any two nodes (except the neighbors) were connected by an edge if the distance between any pair of atoms of the respective residues was less than 4 Å for more than 75% of the simulation time. Covariance and correlation matrices for dynamical network analysis were calculated with the Carma program^35^. Molecular dynamic simulations with QM/MM potentials were performed as described in^21^ for mSAASoti, M163A and F177S variants in their green form. The length of each MD trajectory was 10 ps.

### Protein crystallization

An initial crystallization screening of C21N mSAASoti was performed with a robotic crystallization system (Oryx4, Douglas Instruments, UK) and commercially available 96-well crystallization screens (Hampton Research, Aliso Viejo, CA, USA) at 15 °C using the sitting drop vapor diffusion method. The protein concentration was 5.7 mg/mL in the following buffer: 20 mM Tris, pH 7.5. Crystals used for x-ray experiment were obtained within ~ 1 month in the following conditions: 0.1 M Tris pH 8.0, 30% w/v Polyethylene glycol monomethyl ether 2000.

### Data collection, processing, structure solution and refinement

The diffraction data were collected from a single crystal at 100 K using the X-ray diffractometer Rigaku XtaLAB Synergy-S (Rigaku, USA). The data were indexed and integrated using XDS program^36^ and scaled with Aimless^37^ (Table S2). The structure was solved by the molecular replacement using MOLREP program^38^ and structure of the fluorescent protein Dronpa (PDB ID—6NQP) as an initial model. The refinement of the structure was carried out using Refmac5^39^ implemented in the CCP4 suite. The visual inspection of electron density maps and the manual rebuilding of the model were carried out using COOT^40^. The ProSMART protocol with structure of Dronpa fluorescent protein as a reference model as well as TLS and NCS restraints were used during the refinement. In the final model an asymmetric unit contained two protein copies of 218 residues each, two chromophores and four solvent molecules. The atomic coordinates and structure factors have been deposited in the Protein Data Bank with PDB ID—8PEI.

### Supplementary Information

Supplementary Information.

## Data Availability

The datasets used and/or analyzed during the current study are available from the corresponding author on reasonable request.
