# Supplementary material for: The role of the correlated motion(s) of the chromophore in photoswitching of green and red forms of the photoconvertible fluorescent protein mSAASoti
Source: Sci Rep. 2024 Apr 16;14:8754. doi: 10.1038/s41598-024-59364-1 (PMC11021400; doi:10.1038/s41598-024-59364-1)
Supplement: Supplementary file 1 — Supplementary Information. [file 41598_2024_59364_MOESM1_ESM.docx]

**Supplementary Material**

**The role of the correlated motion(s) of the chromophore in photoswitching of green and red forms of the photoconvertible fluorescent protein mSAASoti**

Alexandra V. Gavshina1, Ilya D. Solovyev1, Maria G. Khrenova1,2,3, Konstantin M. Boyko1, Larisa A. Varfolomeeva1, Mikhail E. Minyaev4, Vladimir O. Popov1, Alexander P. Savitsky1*

1A.N. Bach Institute of Biochemistry, Federal Research Centre ‘Fundamentals of Biotechnology’ of the Russian Academy of Sciences, Moscow, Russia

2Department of Chemistry, M.V. Lomonosov Moscow State University, Moscow, Russia

3Emanuel Institute of Biochemical Physics, Russian Academy of Sciences, Moscow, Russia

4N.D. Zelinsky Institute of Organic Chemistry Russian Academy of Sciences, Moscow, Russia

*Corresponding author

**Table S1.** Multiple sequence alignment of mSAASoti with mIrisFP and Dronpa. The chromophore is colored in green, the a.a. residues mutated in the study are highlighted in purple.

| mSAASoti mIrisFP_2VVH Dronpa_2IE2 | MALSKQYIPDDMELIFHMDGCVNGHYFTIVATGKAKPYEGKQNLKATVTKGAPLPFSTDI 60  ----MSAIKPDMKINLRMEGNVNGHHFVIDGDGTGKPFEGKQSMDLEVKEGGPLPFAFDI 56  ----MSVIKPDMKIKLRMEGAVNGHPFAIEGVGLGKPFEGKQSMDLKVKEGGPLPFAYDI 56  . * **:: ::*:* **** *.* . * .**:****.:. *.:*.****: ** |
| --- | --- |
| mSAASoti mIrisFP_2VVH Dronpa_2IE2 | LSTVMHYGNRCIVHYPPGIPDYFKQSFPEGYSWERTFAFEDGGFCTVSADIKLKDNCFIH 120  LTTAFHYGNRVFAEYPDHIQDYFKQSFPKGYSWERSLTFEDGGICIARNDITMEGDTFYN 116  LTTVFCYGNRVFAKYPENIVDYFKQSFPEGYSWERSMNYEDGGICNATNDITLDGDCYIY 116  *:*.: **** :..** * ********:******:: :****:* . **.:..: : |
| mSAASoti mIrisFP_2VVH Dronpa_2IE2 | TSMFHGTNFPADGPVMQRKTIQWEKSIEKMTVSDGIVKGDITMFLLLEGGGKYRCQFHTS 180  KVRFHGVNFPANGPVMQKKTLKWEPSTEKMYVRDGVLTGDITMALLLEGNAHYRCDSRTT 176  EIRFDGVNFPANGPVMQKRTVKWEPSTEKLYVRDGVLKGDVNMALSLEGGGHYRCDFKTT 176  *.*.****:*****::*::** * **: * **::.**:.* * ***..:***: :*: |
| mSAASoti mIrisFP_2VVH Dronpa_2IE2 | YKAK-KVVEMPQSHYVEHSIERTNDDGT--QFELNEHAVARLNEI----- 222  YKAKEKGVKLPGYHLVDHCIEILSHDKDYNKVKLYEHAVAHSGLPDNARR 226  YKAK-KVVQLPDYHFVDHHIEIKSHDKDYSNVNLHEHAEAHSELPRQAK- 224  **** * *::* * *:* ** ..* :.:* *** *: |

**The example of the pKa value calculating**

pKa values were calculated using modified Henderson-Hasselbalch equation as described in (Solovyev et al. 2018) according to equation S1:

$$H^{+}+{Chrom}^{-}\underset{\Leftrightarrow}{K} ChromH$$

$I\left( \left[ H^{+} \right] \right)=\frac{I_{0}*K}{K+[H^{+}]}$+c (S1)

where *I* correspond to fluorescence intensity at current pH value, *I_0_* – maximum fluorescence intensity at higher pH (anionic chromophore form), K – equilibrium constant, [H+] – current equilibrium proton concentration, c – background including residual intensity of protonated chromophore state. Data analysis was performed by fitting of experimental data according to formula (S1) using Origin 8.5 software package.

| **M163A** | | | **C21N/M163G** | | |
| --- | --- | --- | --- | --- | --- |
| Reduced Chi-Sqr | 239.014 | | Reduced Chi-Sqr | 8.099 | |
| Adj. R-Square | 0.989 | | Adj. R-Square | 0.997 | |
|  | Value | SE |  | Value | SE |
| I_0_ | 343.8 | 11.3 | I_0_ | 148.5 | 1.9 |
| K | 2.236E-7 | 3.64E-8 | K | 9.37E-8 | 5.12E-9 |
| c | 5.28 | 8.17 | c | 0 | - |


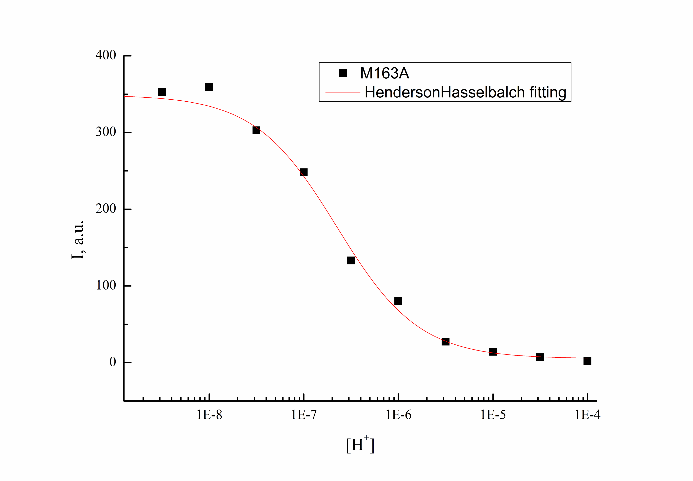

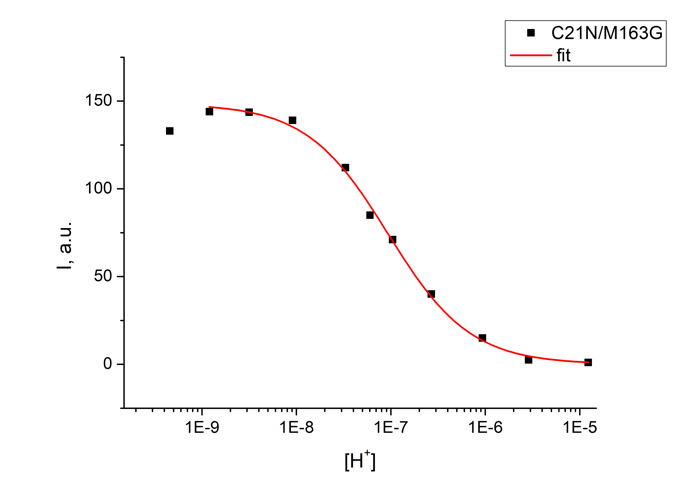


**Figure S1.** Dependence of the fluorescence intensity on the pH value measured for pK calculation for the green M163A and F177S mSAASoti mutants.


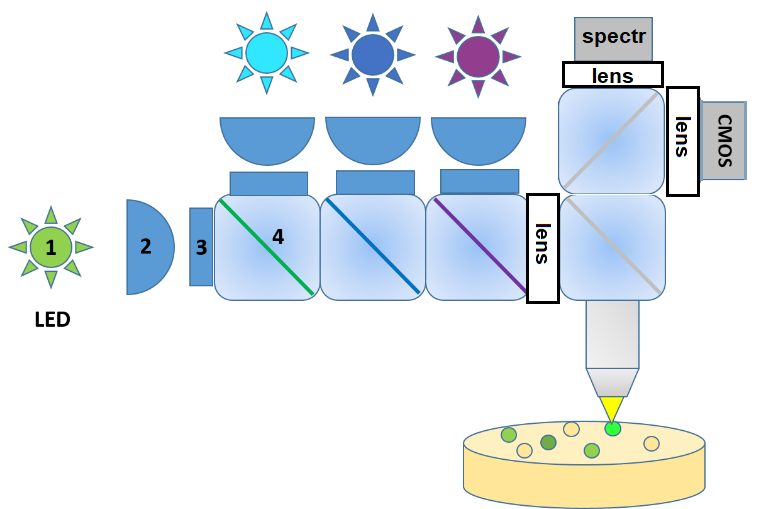

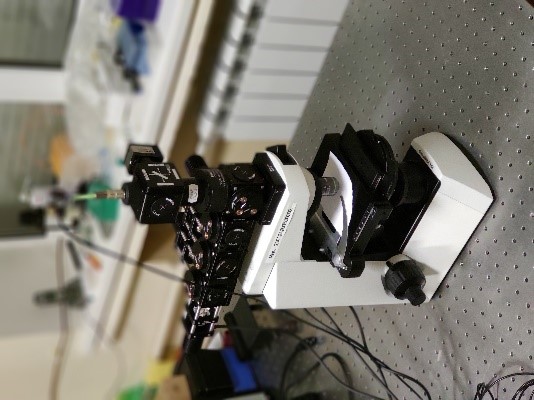


**Figure S2**. Optical scheme and photo of the photochemical screening setup. 1) LEDs; 2) condenser lenses; 3) bandpass filters; 4) dichroic mirror.

Green-to-red photoconversion is usually accompanied with the parallel photodestruction processes. To neutralize the effect of photochemical changes that would affect the red form, we examined a partially converted protein (in solution). The exposure dose of 400 nm light was chosen in such a way as to obtain the maximum intensity of the red form. The fact is that in such conditions even in the wild type protein, irradiation with 400 nm light does not lead to a complete green-to-red photoconversion (Figure S3 and S4), there is always a mixture of the forms (green and red). M163X and F177X substitutions, indeed, can also affect the conversion efficiency, however, improving the photoconversion was not the goal of this study. Since the photoconversion of SAASoti proceeds according to the Kaede-mechanism including a photoinduced break of the polypeptide chain, the degree of photoconversion can also be estimated as the ratio of the products before and after the illumination. The samples before and after 400 nm (10 min) irradiation were also analyzed by Laemmley gel electrophoresis (Figure S4). According to the results calculated from the gel-electrophoresis, the degree of the peptide chain break in the case of C21N/M163T seems to be less compared to the wt and F177S variants. Several reasons can be assumed, however, requiring separate study. One of the possible reasons for the phenomenon are the PC rate, the quantum yield and pH-dependency of the process. Previously (Gavshina A.V. et al, Sci Rep 11, 24314, 2021) we studied the photoconversion rate during 400 nm illumination by recording the emission spectra of the red form in time when irradiated with light of 400 nm, however, it was also necessary to turn on the 550 nm light (low power) in order to excite the red form. In the case of the M163X and F177X mutant variants, it is difficult to implement, since irradiated with the green light red form can be simultaneously and rapidly switched-off to the dark state.

**A B**


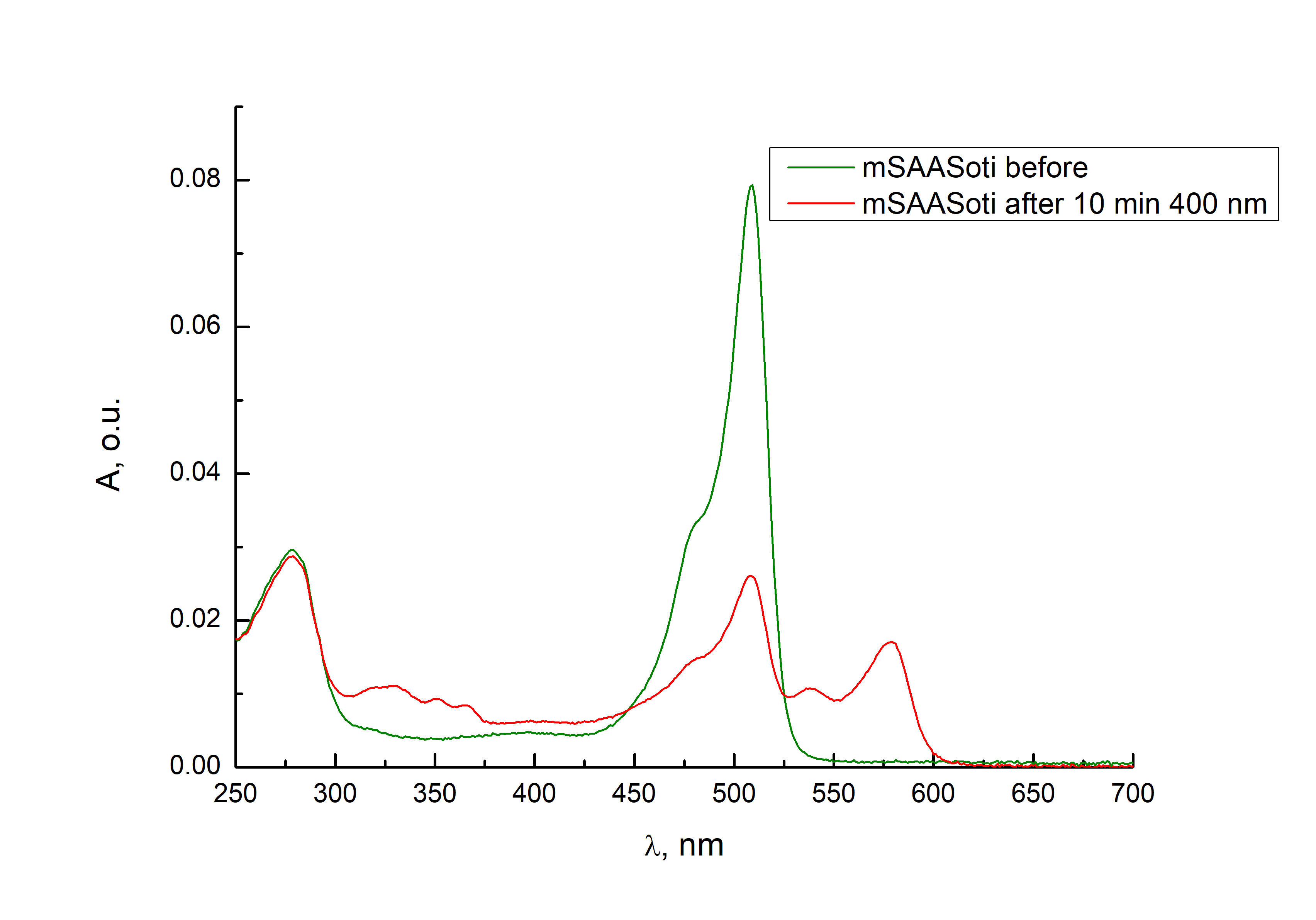

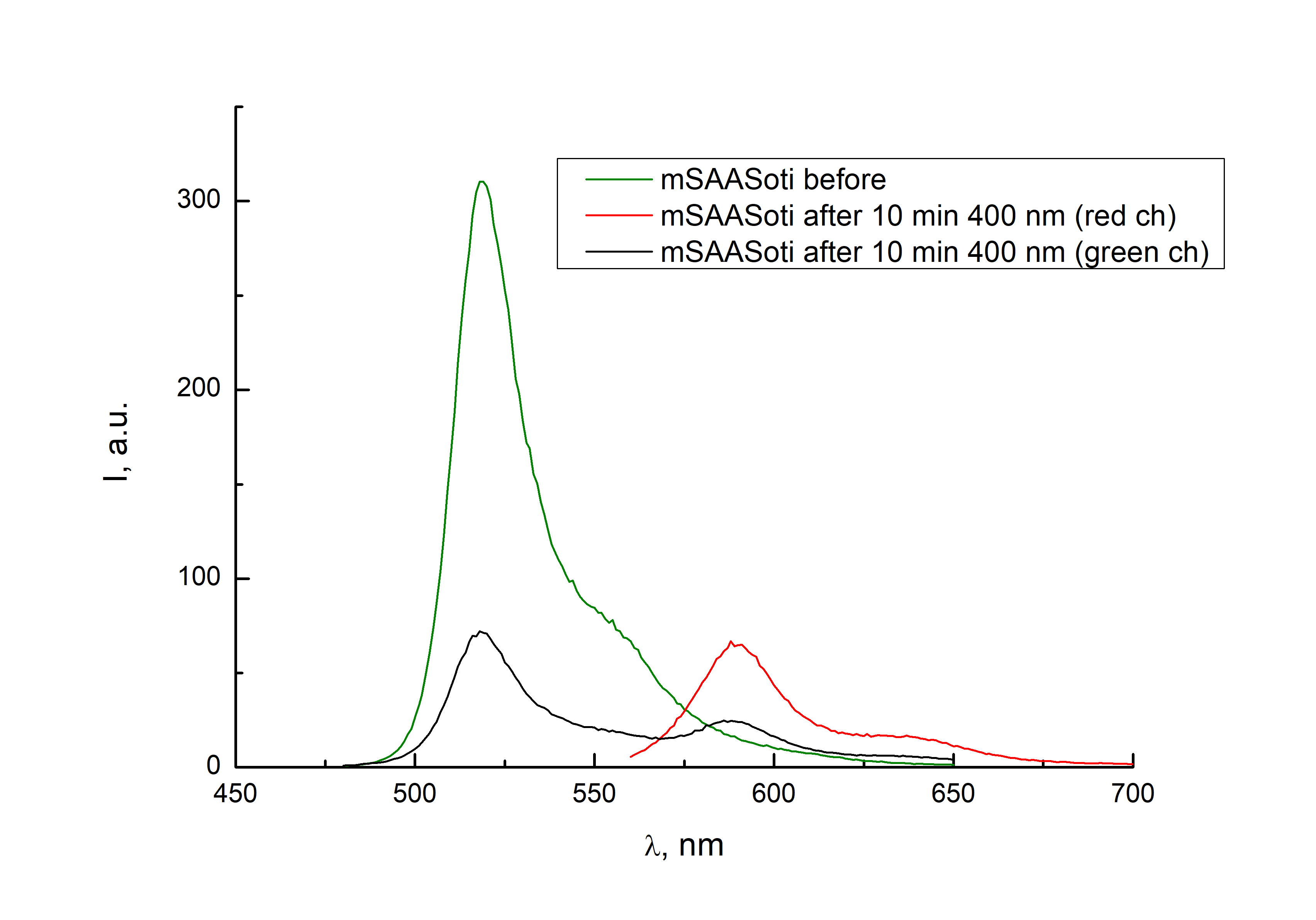


**C D**


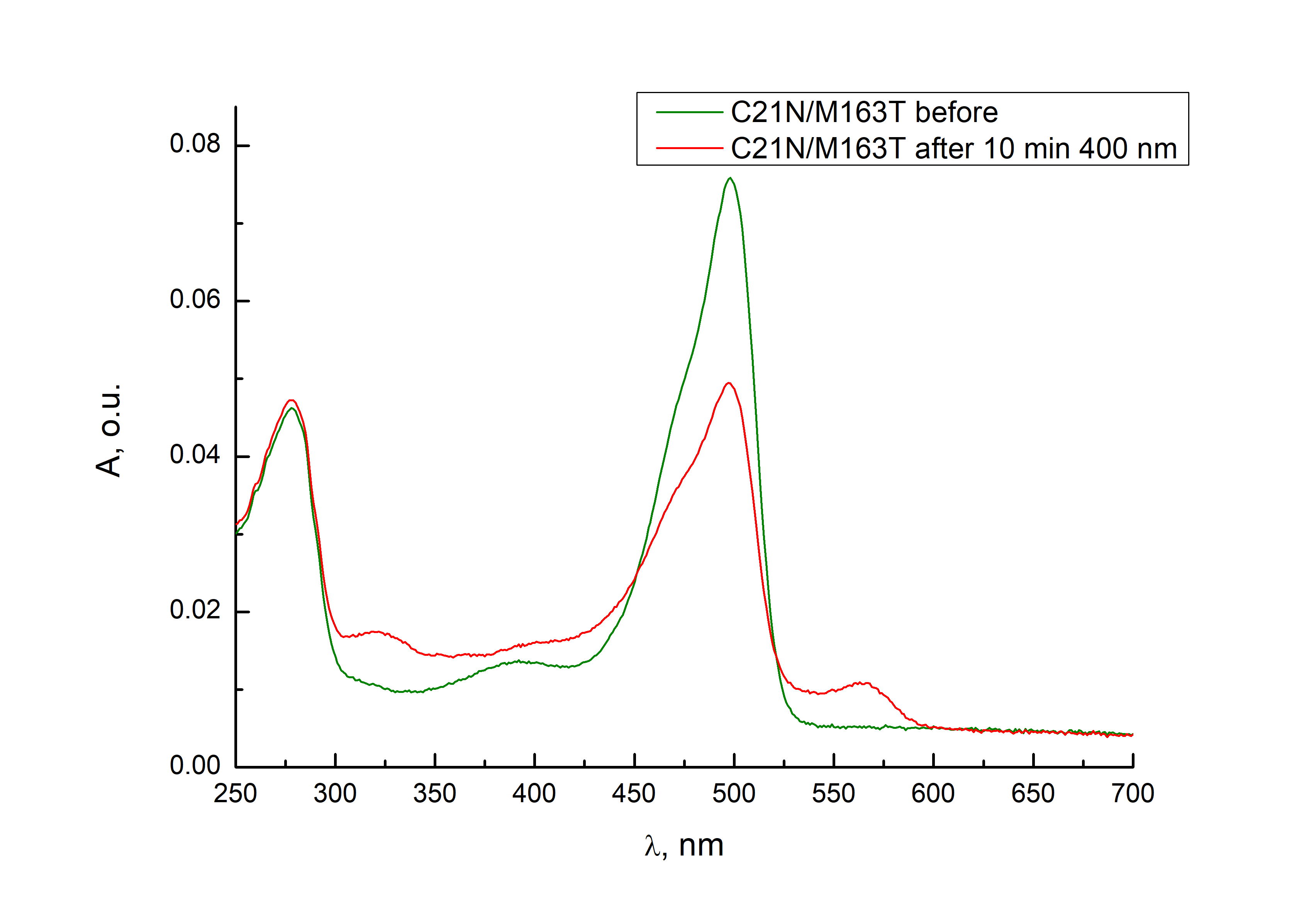

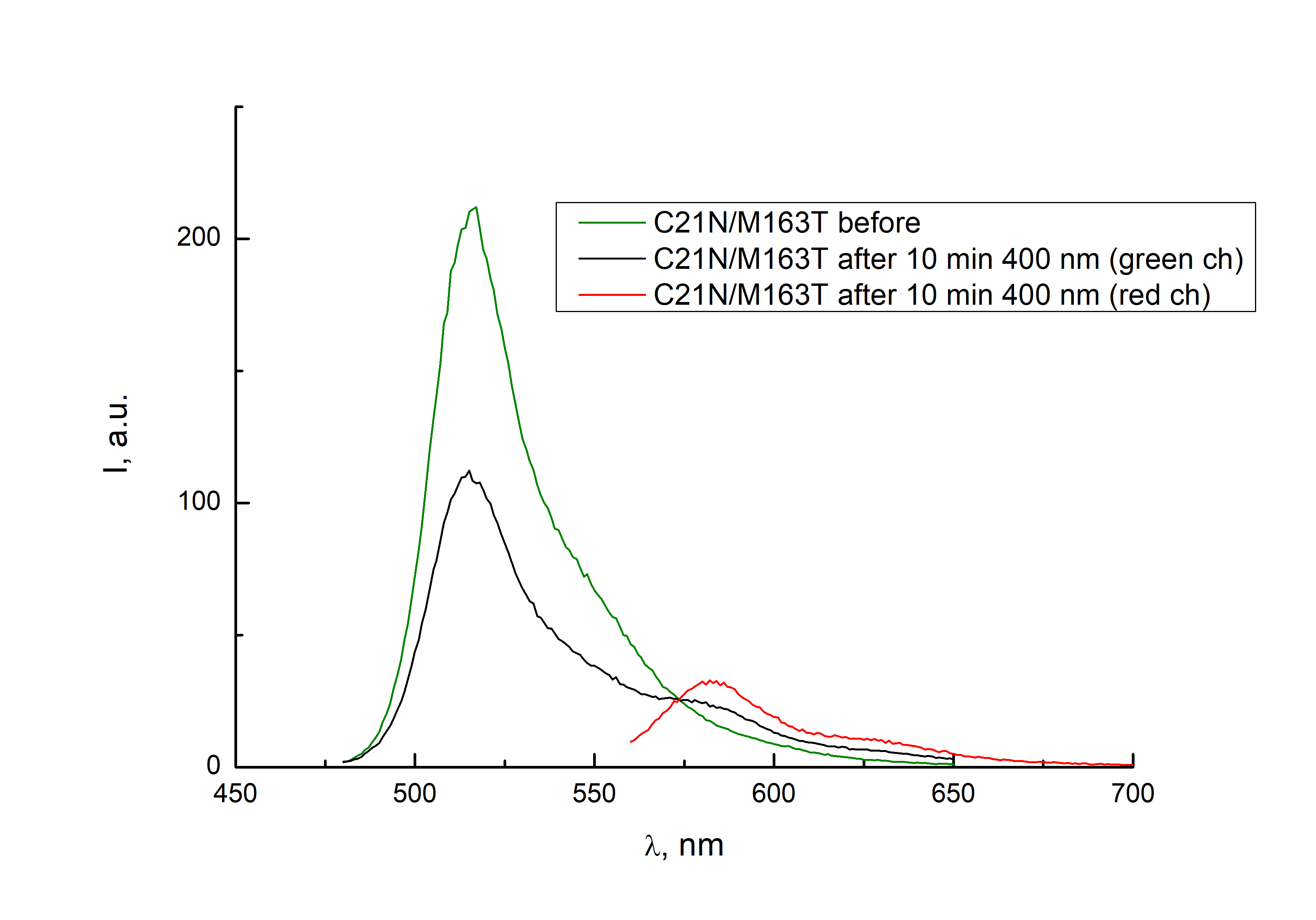


**E F**


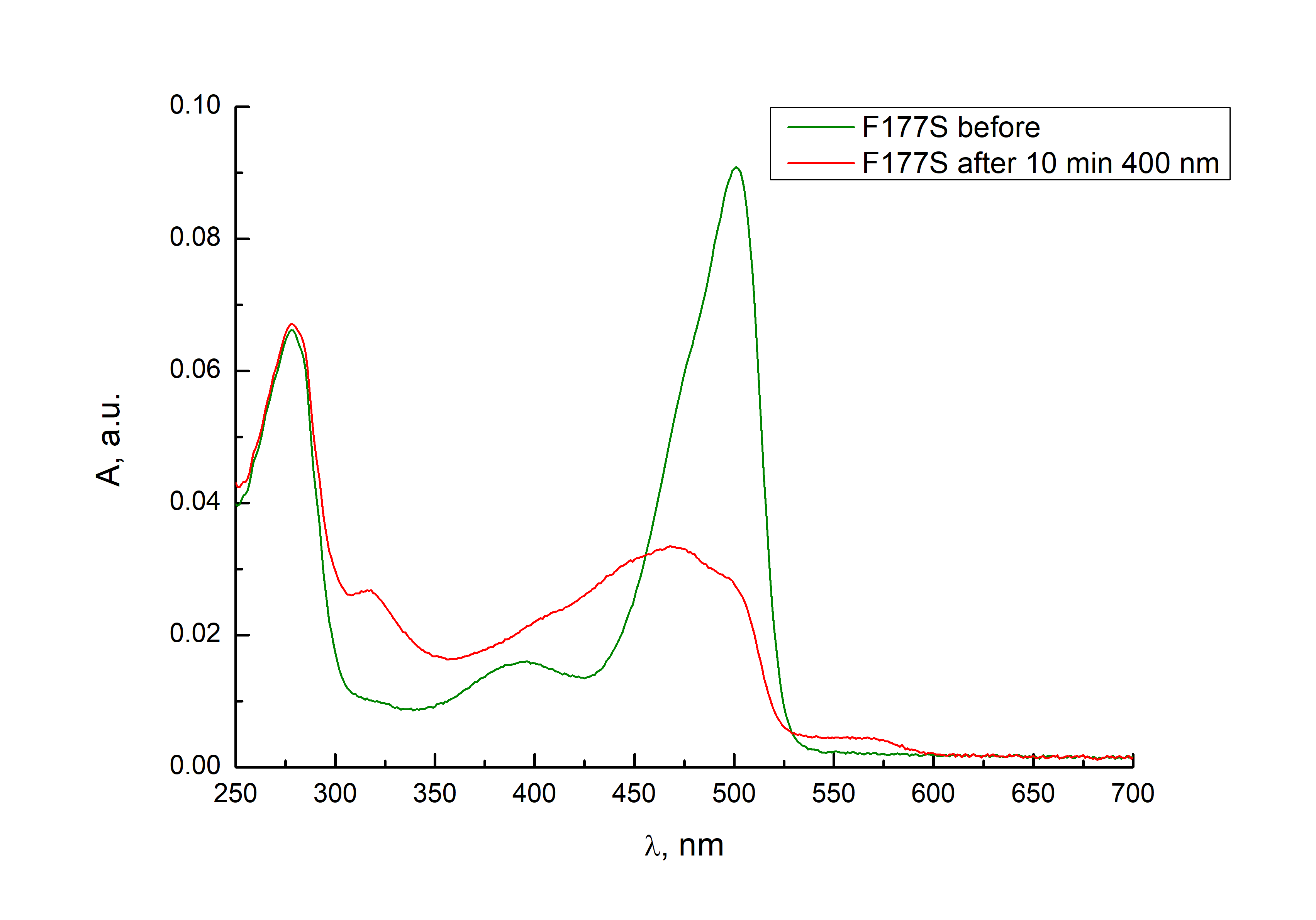

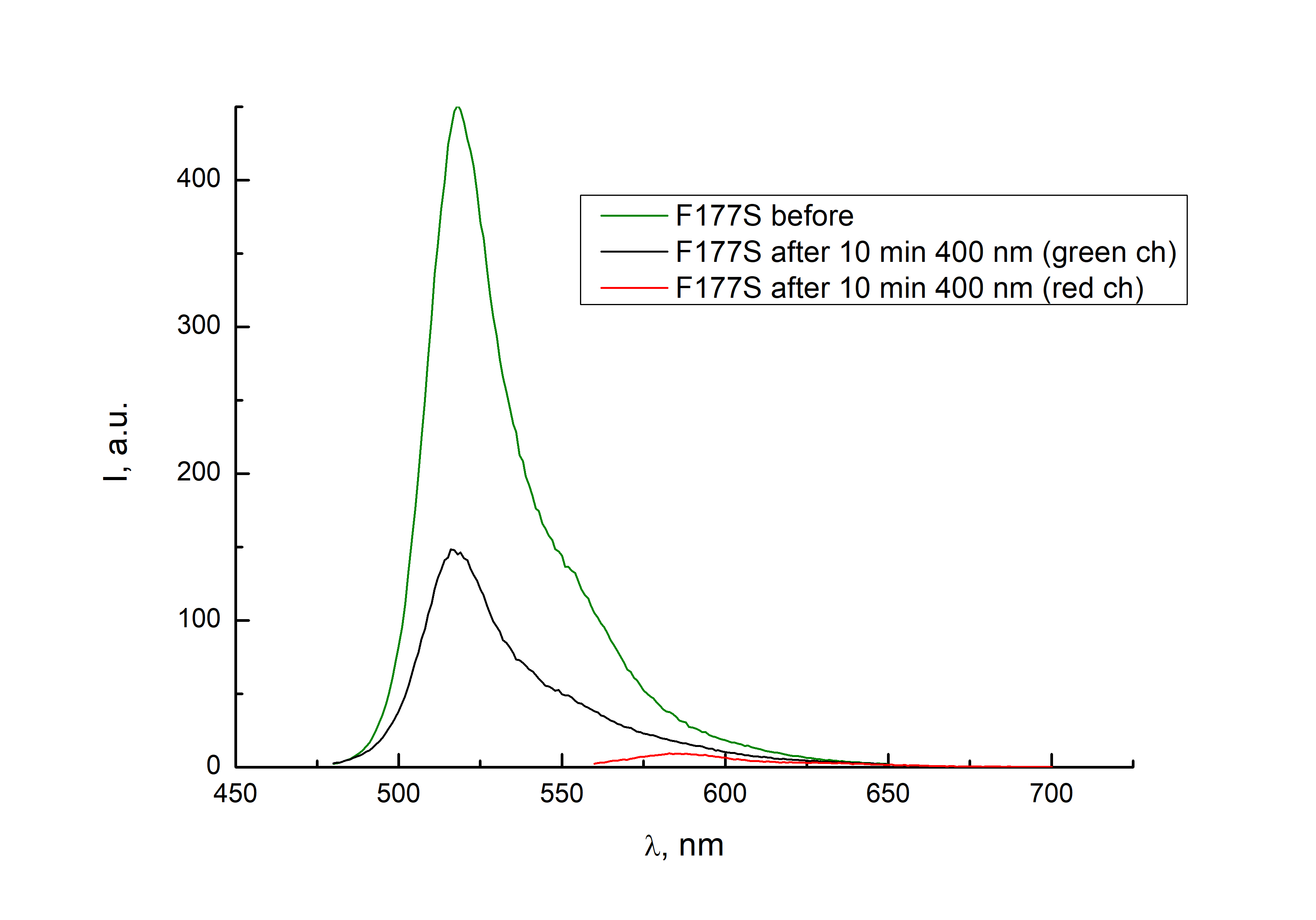


**Figure S3.** Absorbance (A, C, E) and emission (B, D, F) spectra of mSAASoti, C21N/M163T and F177S variants before (green line) and after (red and black lines) 400 nm light illumination during 10 min measured in 200 mM Tris-HCl buffer pH 7.4 on Cary 60 and Cary Eclipse. Green and red forms were excited with 470 (green channel) and 550 nm (red channel) light, respectively.

| mSAASoti | % cleavage |
| --- | --- |
| wt | 55 |
| C21N/M163T | 28 |
| F177S | 55 |


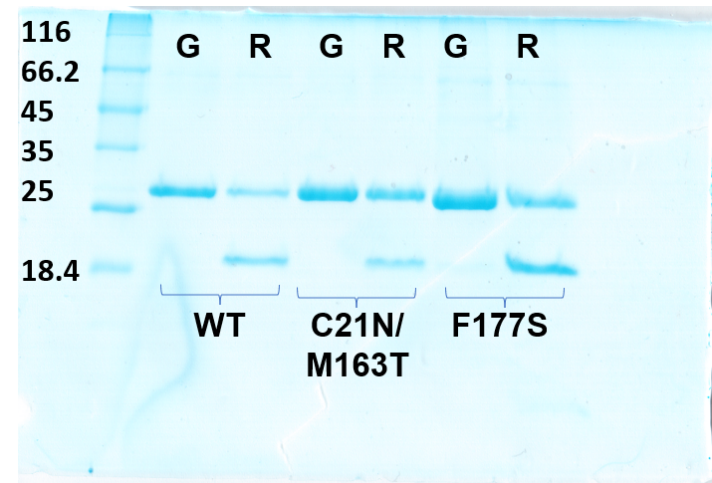


**Figure S4.** A Page-blue stained SDS polyacrylamide gel of mSAASoti mutants before (G, green) and after (R, red) 400 nm illumination during 600 s. The lower band in R-lines occurs as a result of the photoinduced peptide bond cleavage during green-to-red photoconversion under 400 nm illumination (Kaede-type photoconversion). The table shows % of the peptide chain cleavage calculated using the ImageJ program.

**A B**


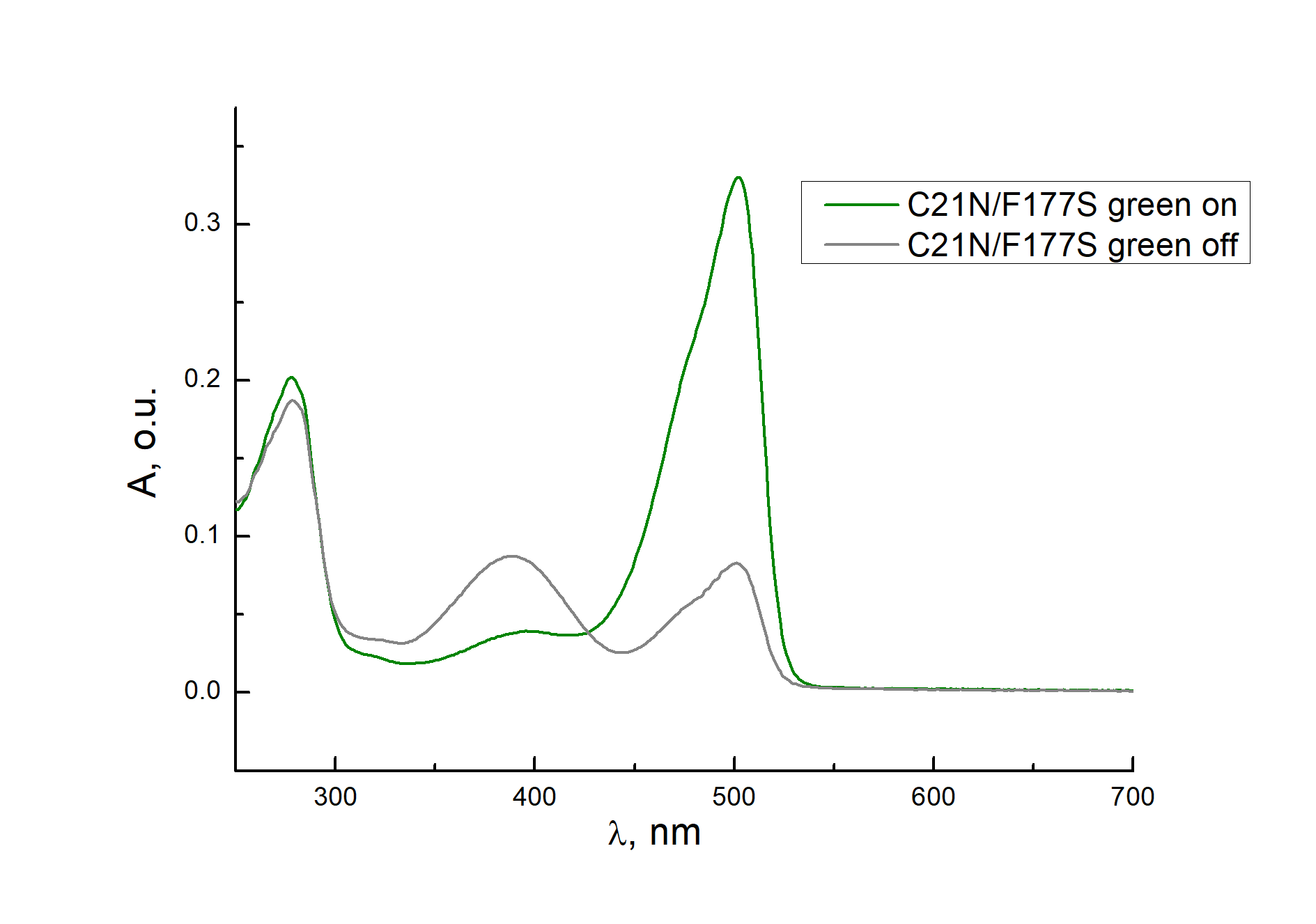

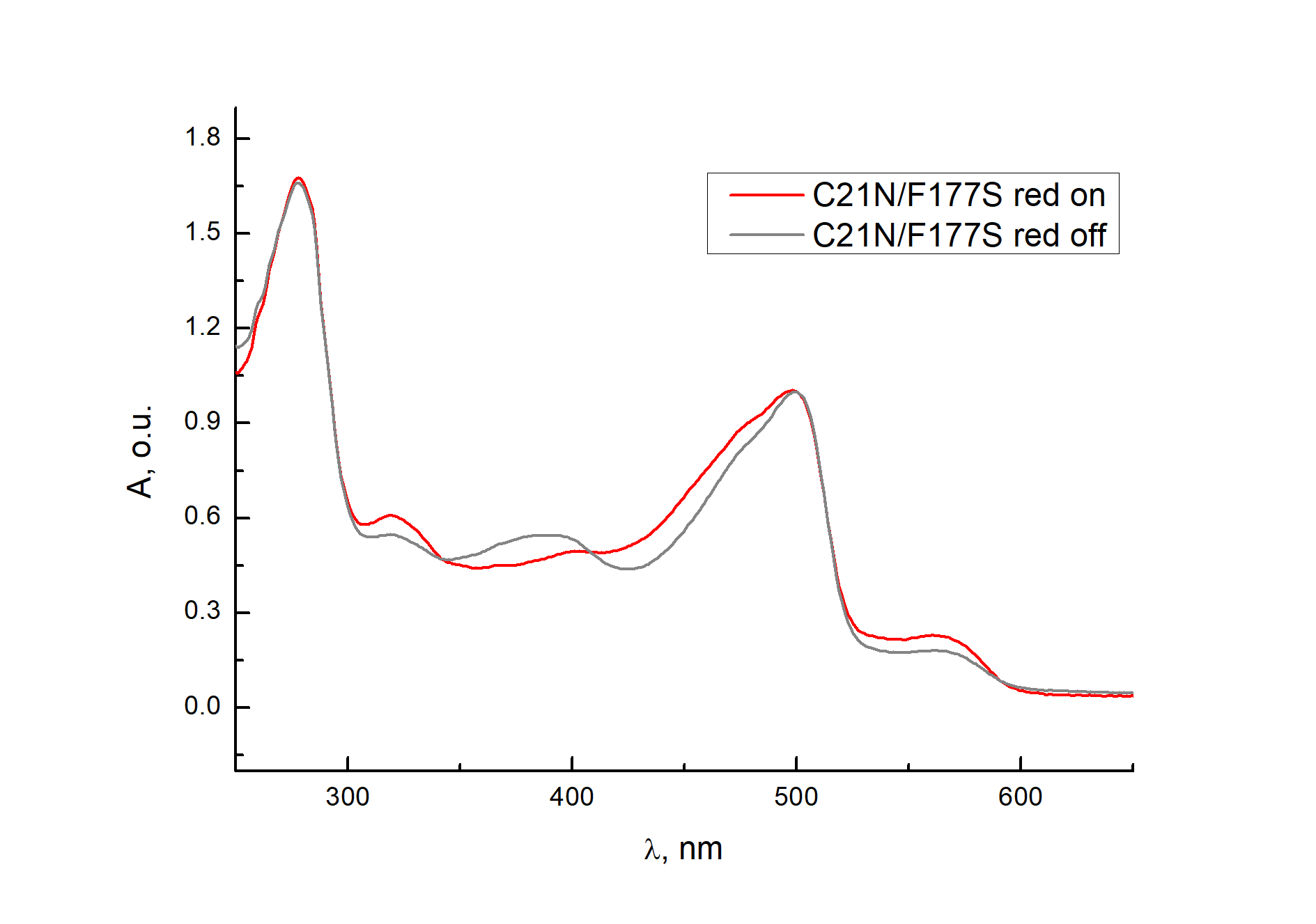


**C D**


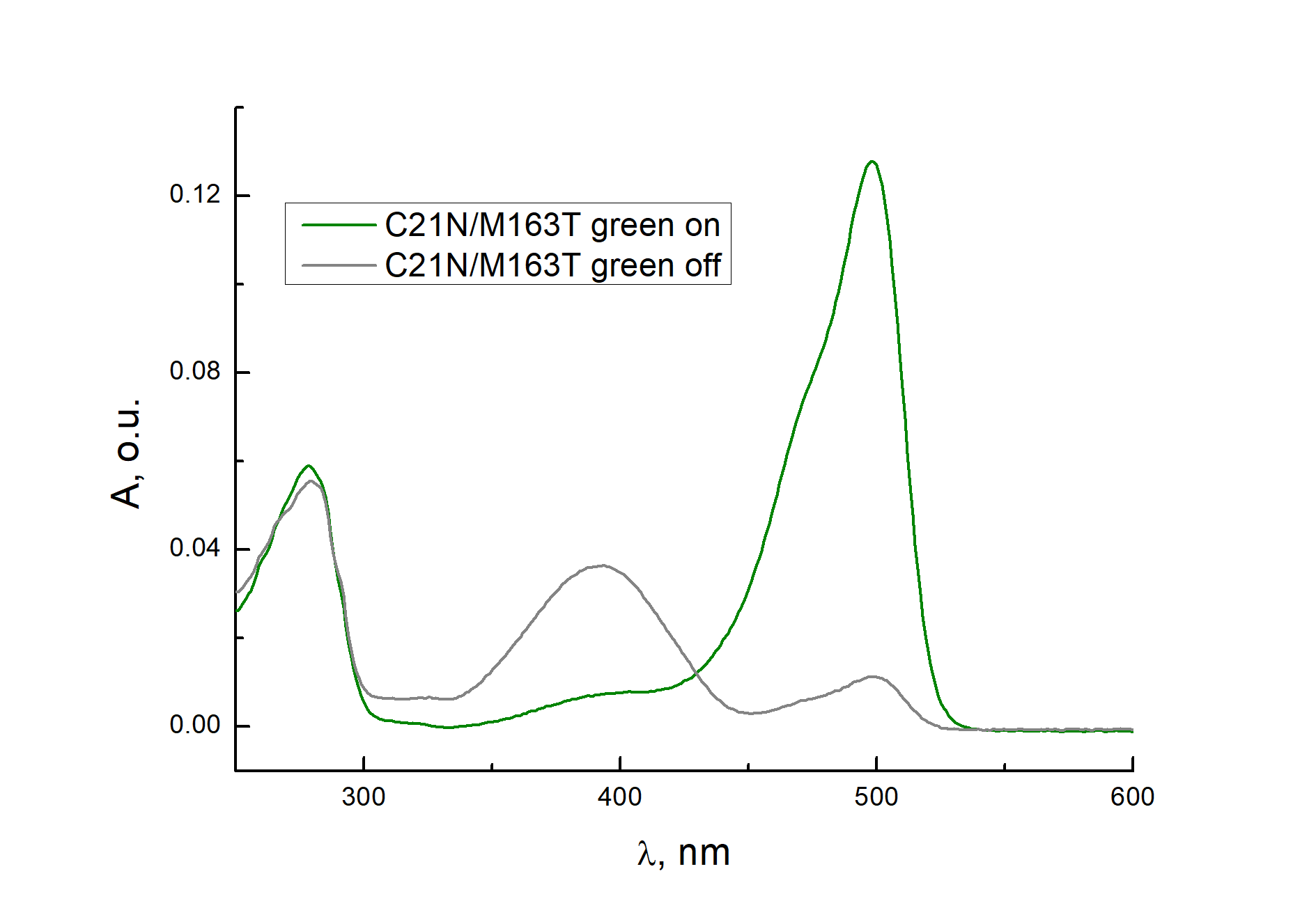

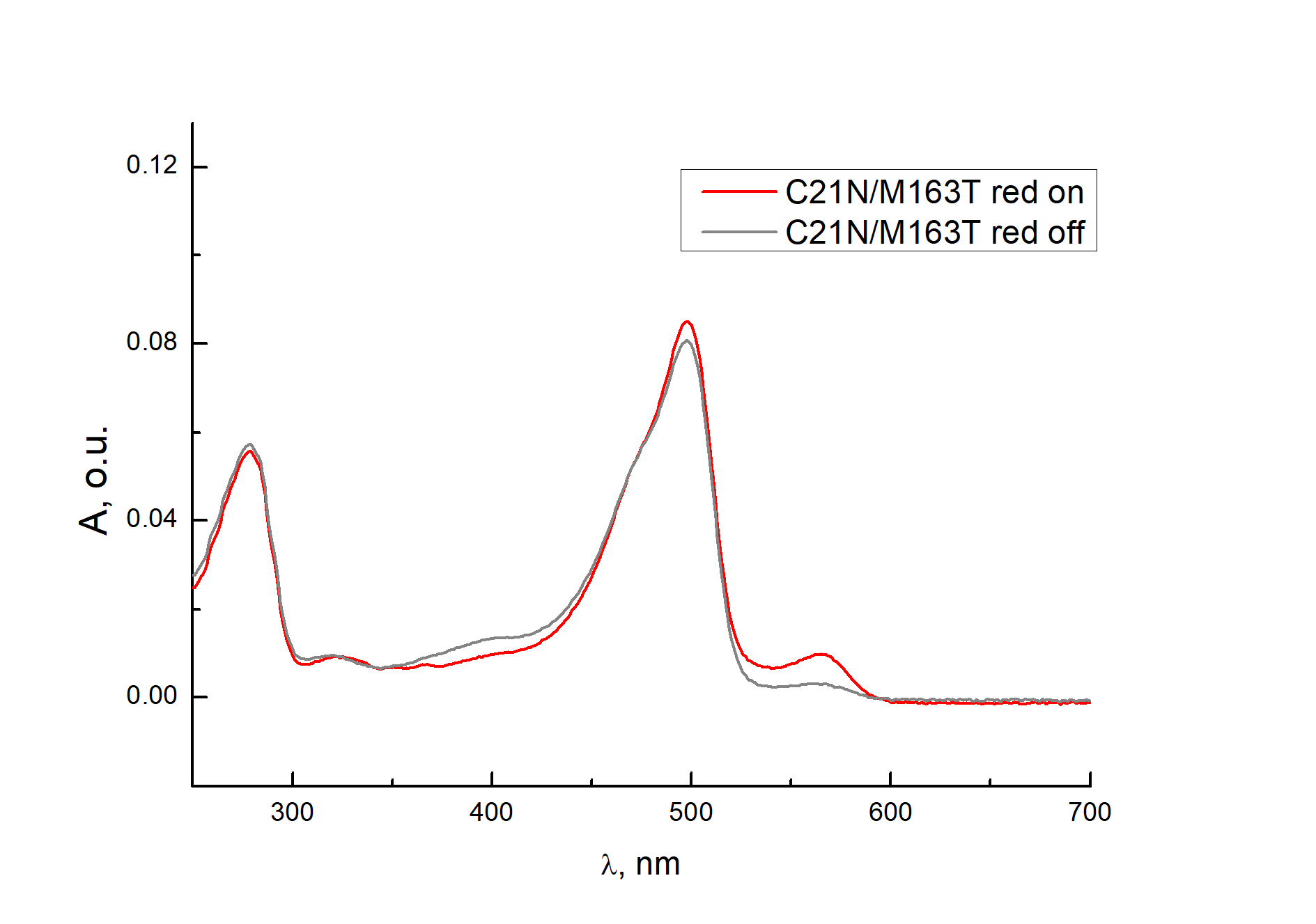


**Figure S5.** Absorbance spectra of the green and red C21N/F177S (A, B) and C21N/M163T (C, D) mSAASoti variants measured in 200 mM phosphate buffer pH 7.5 on Cary 60. On-to-off PS of the green form was realized by illumination the cuvette with 485 nm light during 200 s, and on-to-off PS of the red form was performed by 550 nm illumination during 600s. Green-to-red PC was performed prior to PS of the red-form by illumination the green sample with 400 nm light during 600 s.

**Table S2.** Data collection, processing, and refinement.

| **Data Collection** | |
| --- | --- |
| Diffraction source | Rigaku XtaLAB Synergy-S |
| Wavelength (Å) | 1.54 |
| Temperature (K) | 100 |
| Detector | HyPix 6000HE |
| Crystal-to-detector distance (mm) | 53.0 |
| Rotation range per image (°) | 0.5 |
| Total rotation range (°) | 250 |
| Space group | P6_1_22 |
| a, b, c (Å) | 104.58; 104.58; 151.19 |
| α, β, γ (°) | 90.0; 90.0; 120.0 |
| Unique reflections | 10148 (1516) |
| Resolution range (Å) | 24.28-3.0 (3.18-3.0) |
| Completeness (%) | 98.0 (92.6) |
| Average redundancy | 26.9 (28.8) |
| 〈*I*/σ(*I*)〉 | 7.1 (1.1) |
| Rpim (%) | 2.8 (88.4) |
| CC_1/2_ | 98.5 (60.1) |
| **Refinement** | |
| R_fact_ (%) | 29.1 |
| R_free._ (%) | 34.0 |
| Bonds (Å) | 0.01 |
| Angles (°) | 2.05 |
| **Ramachandran plot** |  |
| Most favored (%) | 96.4 |
| Allowed (%) | 3.6 |
| **No. atoms** |  |
| Protein | 3413 |
| Water | 4 |
| Chromophore | 50 |
| **B-factors (Å^2^)** |  |
| Protein | 67.71 |
| Water | 16.85 |
| Chromophore | 54.15 |
| Molprobity score | 2.62 |
| PDB ID | 8PEI |

Values in parenthesis are for the highest-resolution shell
